# Ceramic Piezoelectric Transformer in Vacuum for Acceleration of Electrons and Production of X-Rays

**DOI:** 10.3390/ma11071188

**Published:** 2018-07-11

**Authors:** Alexander V. Shchagin, Viktor S. Miroshnik, Vladimir I. Volkov, Aleksandr S. Kubankin, Oleg O. Ivashchuk

**Affiliations:** 1 International Scientific and Educational Laboratory of Radiation Physics, Belgorod State National Research University, Belgorod 308015, Russia; kubankin@bsu.edu.ru (A.S.K.); oleg.iwaschuck@yandex.ru (O.O.I.); 2 National Science Center Kharkov Institute of Physics and Technology, Kharkov 61108, Ukraine; miroshnik@kipt.kharkov.ua (V.S.M.); av.shchagin@gmail.com (V.I.V.); 3 P.N. Lebedev Physical Institute RAS, Moscow 119991, Russia

**Keywords:** ceramic piezoelectric transformer, vacuum, accelerator, X-ray radiation

## Abstract

Experiments on acceleration of electrons and production of X-ray radiation with use of ceramic piezoelectric transformers installed in vacuum are described and analyzed. The piezoelectric transformer operates at resonance frequency. Electrons are accelerated from the high-voltage electrode of the ceramic piezoelectric transformer toward the grounded target, where they emit bremsstrahlung and characteristic X-ray radiation in the target material. The returning of the charge to the high-voltage electrode is provided due to electrons emitted from a filament installed in the vicinity of the target. It was found that the X-ray yield increases linearly at increasing of the pressure of the residual gas in the chamber within two orders of magnitude up to about 10 mTorr, when the gas discharge around of the piezoelectric transformer arises. Possibilities for application of piezoelectric transformers for production of accelerating voltage in small-size accelerators are discussed.

## 1. Introduction

Usually, X-ray radiation is produced by charged particles accelerated by one of the classic methods of acceleration [1]. However, more recently, a few new methods of particle acceleration up to the energies of about 100 keV in miniature accelerators without any outer high-voltage power supply have been proposed. For instance, X-ray radiation is produced by electrons accelerated in small-size accelerators in the electric field arising in vacuum due to the pyroelectric [2,3,4], triboelectric [5,6] or piezoelectric [7] effects and also in the high-frequency field which is generated by a piezoelectric transformer in vacuum. The research with crystalline piezoelectric transformer installed in vacuum was performed in [8] (see also [9,10]). Recently, observation of X-rays with application of ceramic transformer in vacuum was first reported in the short communication [11]. Here, the experimental research on acceleration of electrons and production of X-rays with use of ceramic piezoelectric transformer installed in vacuum is described and possibilities for application of ceramics piezoelectric transformers for production of accelerating voltage in small-size accelerators are discussed.

## 2. Materials and Methods

**Figure 1:**
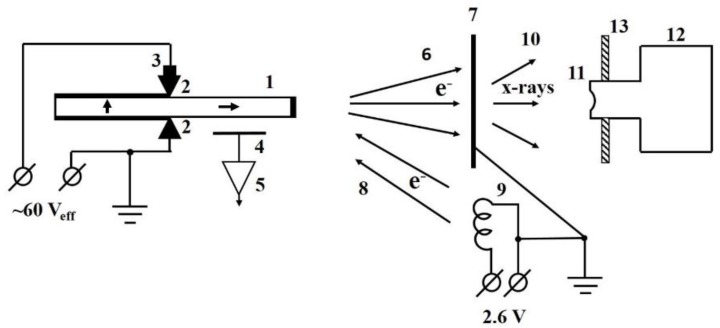
The experimental setup. Ceramic piezoelectric transformer **1** is installed between contacts **2**; two low-voltage and one high-voltage silver electrodes of the piezoelectric transformer are shown by bold lines; one of the contacts is supplied by digital thermometer **3**; the sine signal from the electric field probe **4** penetrates to the input of the amplifier **5**; electrons **6** are accelerated from the high voltage electrode towards a thin target **7** during negative high voltage half-wave; electrons **8** move from the filament **9** towards the high voltage electrode during positive high voltage half-wave; X-rays **10** from the target reach the entrance Be window **11** of the X-ray detector **12**; the entrance widow of the detector is sunk into the vacuum chamber through the flange **13**. Polarization directions are shown by bold arrows in the ceramic piezoelectric transformer bar.

Experiments were performed in the vacuum chamber of inner diameter 250 mm and height 300 mm. The scheme of the experimental setup in the vacuum chamber is shown in Figure 1.

The Rosen type single layer piezoelectric transformer (Monolit, Vitebsk, Belarus) made of ceramics based on lead zirconate titanate was used in the experiment. According to producer’s specification, the basic resonance frequency of the transformer is 19–22 kHz, the input capacity is 1800–2300 pF, the output capacity is 7–9 pF, the output voltage is no less than 3.0 kV and input current no more than 20 mA at input effective voltage 20 V and 100 MΩ loading resistor, the coefficient of efficiency is no less than 0.5 at 30 MΩ loading resistor, and the maximum input voltage is 60 V. The bar size is 80 mm × 10 mm × 3 mm. The piezoelectric transformer was clumped up in vacuum between two spring-loaded contacts in the middle of the bar (see Figure 1) and operated at first harmonic with resonance frequency close to 20 kHz. The power supply of the transformer was provided by an outer generator, which produces the sine signal of effective voltage 60 ± 0.5 V of resonance frequency. The frequency of the generator smoothly tuned to find the resonance frequency of the piezoelectric transformer. No special electron emitter was installed at high voltage electrode of the transformer, but electrons could be emitted from the surface irregularities of the electrode in the electric field between the electrode and grounded target. The target made of 20 µm thick titanium foil was installed at the distance of 30 mm from the high voltage electrode. The filament was installed near the target. The glower of 3.5 V 0.28 A incandescent lamp with removed glass bulb was used as the filament.

To study acceleration of electrons, we observed X-rays excited by accelerated electrons in a thin target. Spectra of X-ray radiation were measured with the X-ray spectrometer (Amptek, Bedford, MA, USA) that consists of the 5 mm × 5 mm × 1 mm CdTe X-ray detector XR-100T and the digital pulse processor PX-4 [12] connected to a laptop. The peaking time of the spectrometer was 4 μs. The X-ray spectrometer was energy calibrated with radioactive sources ^55^Fe and ^241^Am. The energy resolution of the X-ray spectrometer (FWHM) was about 420 eV at X-ray energy of 5.9 keV. The 100 µm thick beryllium entrance window of the detector was sunk into the vacuum chamber. The distance between the entrance window and the target was 6 mm.

**Figure 2:**
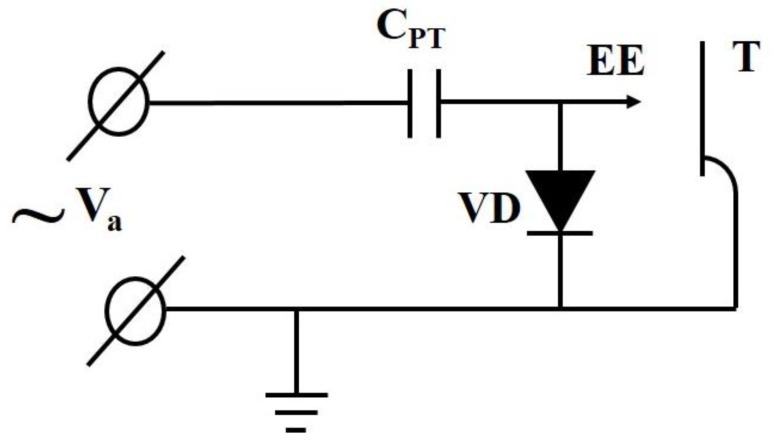
The equivalent scheme of the returning of the charge. The sinusoidal high voltage of amplitude $\;V_{a}$ is produced at the high voltage electrode of the piezoelectric transformer, $\;C_{PT}$ is the output capacity of the piezoelectric transformer, VD is the vacuum diode, EE is the emitter of electrons, and T is the target.

Electrons emitted from the high voltage electrode are accelerated toward grounded target during negative half-wave of voltage at the high-voltage electrode of the transformer. However, the emission of electrons from the high-voltage electrode of the piezoelectric transformer leads to the increasing of average positive potential at the electrode and the reduction of an energy of accelerated electrons [8]. To avoid the reduction, one has to provide returning of a negative charge to the high voltage electrode of the piezoelectric transformer [8]. The application of the vacuum diode for the returning of the charge has been proposed in communication [11]. The equivalent scheme for explanation of the electron current return path is shown in Figure 2.

**Figure 3:**
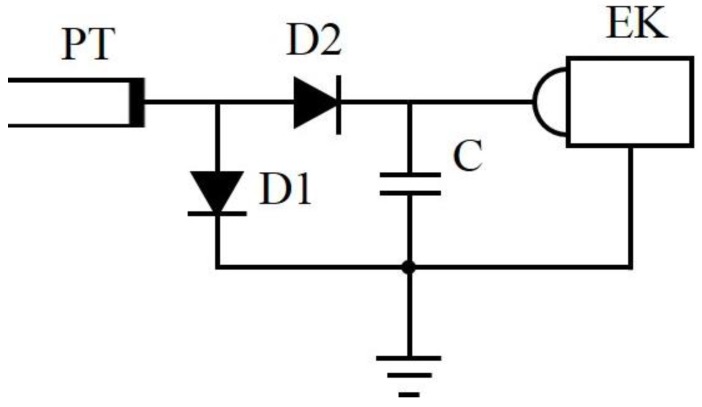
The diagram of the voltage doubler for verification of the value of the high voltage. D1 and D2 are semiconductor 18 kV diodes DD1800, the capacity of the output capacitor C is 100 pF, EK is the electrostatic kilo-Voltmeter with input capacity 9 pF and resistance >> 1 GΩ.

The sinusoidal high voltage of amplitude $V_{a}$ is produced at the high voltage electrode of the piezoelectric transformer with output capacitance $C_{PT}$ and penetrates to the vacuum diode VD and electron emitter EE. The surface irregularities at the high-voltage electrode of the ceramic piezoelectric transformer were used as electron emitters in our experiment. The cathode of the vacuum diode VD is the grounded filament (see Figure 1). The filament emits electrons and provides the electron current to high voltage electrode of the piezoelectric transformer during the positive half-wave of the voltage. As a result, the negative pulsed sinusoidal voltage arises at the electron emitter. The average value of the voltage is $V_{a}$. The maximum negative voltage 2$V_{a}$ appears at the emitter EE with the frequency of the operation of the piezoelectric transformer. The electrons emitted in the moments of the maximum voltage 2$V_{a}$ are accelerated and strike the grounded target with energy 2*e*$V_{a}$, where *e* is the electron charge. To verify the voltage, well known scheme of the voltage doubler shown in Figure 3 was used.

**Figure 4:**
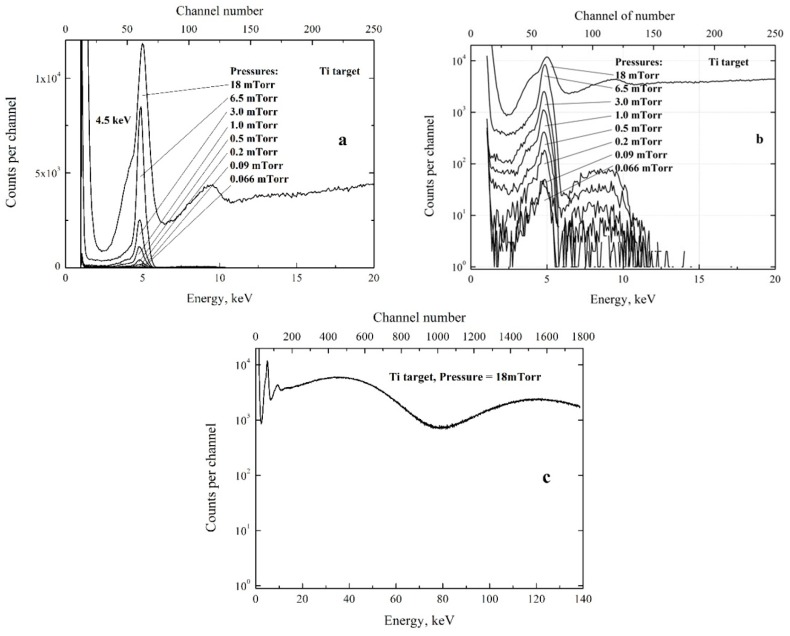
Spectra of X-ray radiation measured at different pressures P of the residual gas are shown in linear (**a**) and logarithmic (**b**) scales as functions of photon energy and corresponding number of channel of the spectrometer. The result of the measurement at P = 18 mTorr is shown separately in the extended energy range (**c**).

The scheme shown in Figure 3 was assembled in the air with semiconductor diodes D1 and D2. The DC positive voltage of value 2$V_{a}$ is produced at the output capacitor C. The piezoelectric transformer operated in the same regime as in vacuum (input AC effective voltage 60 $V_{eff}$ at the resonance frequency about 20 kHz). The measured voltage 12 kV is in agreement with maximum observed energy of X-rays, 12 keV (see Figure 4). It means that the piezoelectric transformer produces the same voltage in air and in vacuum.

The piezoelectric transformer heats at its operation with resonance frequency. However, the temperature regimes of operation of the piezoelectric transformer in vacuum and in air are different. In air, the piezoelectric transformer is cooled mainly by air. In vacuum, the cooling is possible mainly due to thermal radiation and partially through the contacts. The semiconductor digital thermometer DS18B20 was installed on one of the contacts (see Figure 1) to estimate the temperature of the piezoelectric transformer. 

The resonance frequency of the piezoelectric transformer in vacuum somewhat changed in time after turning on because of the heating of the ceramics. Therefore, fine tuning of the generator frequency was executed to provide resonance conditions during measurements of every spectrum.

To control resonance conditions, the electric field probe (cupper strip) was installed at distance of about 1 cm from the high-voltage part of the piezoelectric transformer. The sinusoidal signal from the probe was amplified and observed by an oscilloscope. The resonance frequency was at maximal amplitude of the sine signal from the probe.

## 3. Results

All spectra of X-ray radiation were measured during 5 min at different pressures of the residual gas (air) in the chamber. During every measurement, the temperature measured at one of the contacts of the piezoelectric transformer increased from 35 °C to about 60 °C while the resonance frequency increased by about 150–200 Hz and the amplitude of sine signal from the electric field probe decreased by about 10–15%. The frequency of the generator was smoothly tuned during the measurements to provide maximum signal from the electric field probe and resonance conditions as described above. The spectra measured at different pressures of the residual gas in the chamber are shown in Figure 4.

From the beginning, let us consider all spectra shown in Figure 4a,b measured at the residual gas pressure 0.066 ≤ P ≤ 6.5 mTorr (0.0088 ≤ P ≤ 0.87 Pa). The spectra have the same shape but the yields are different. Every spectrum has spectral peak at energy 4.5 keV with background of bremsstrahlung radiation. The peak energy corresponds to the $K_{\alpha }$ line of titanium atoms composing the target. The sharp decrease of the background yield at X-ray energies some exceeding 5.0 keV corresponds to the sharp increase of the attenuation of X-rays in the Ti target at energies some exceeding the K-edge of attenuation of Ti atoms at 5.0 keV. The maximum energy of the bremsstrahlung is about 12 keV in all spectra. The maximum energy of accelerated electrons should some exceed the maximum energy of the bremsstrahlung. This means that X-rays are produced in the target by electrons with maximum energy close to 12 keV. The maximum energy of the accelerated electrons of 12 keV is practically independent of the pressure.

One can see strong increase of the number of counts in the high-energy part of the spectrum measured at pressure 18 mTorr (2.4 Pa). During this measurement, the appearance of visible light around of the piezoelectric transformer was observed through the glass window of the chamber. The emission of the light was due to gas discharge in the pulsed electric field in the vicinity of the piezoelectric transformer. The acoustic vibration from the piezoelectric transformer could pass through the discharging gas and reach the detector. Besides, similar spectra were observed at much lower pressures if the piezoelectric transformer was shifted and contacts were not exactly in the middle of the transformer bar, where the node of transformer at first harmonic is located. In this case, the acoustic vibrations passed from the piezoelectric transformer through the contacts, the holder of the contacts, and the vacuum chamber walls to the detector. The appearance of the acoustic vibrations in the detector was observed with use of the oscilloscope connected to the exit of the preamplifier of the detector in the view of the 20 kHz sinusoid. Considering the significant sensitivity of CdTe detectors to the acoustic noise, one can conclude that high-energy part of the spectrum measured at 18 mTorr arises not due to X-rays but because of the acoustic noise from the piezoelectric transformer.

**Figure 5:**
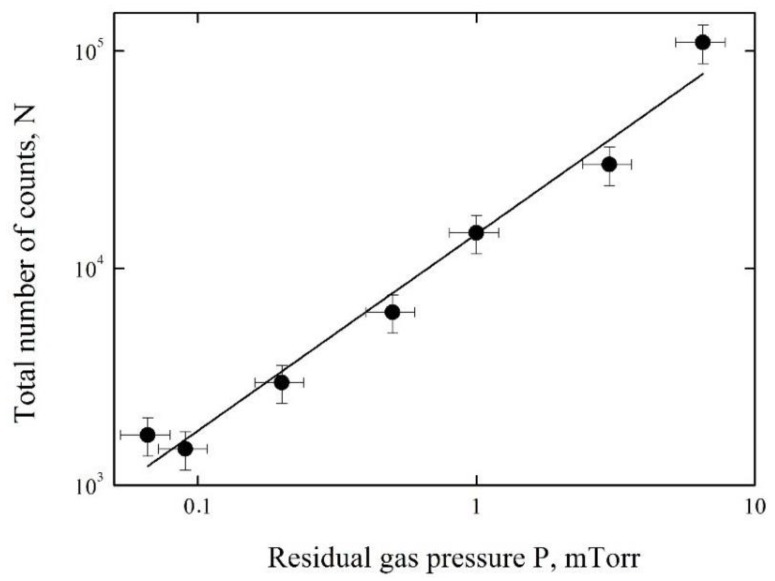
The X-ray yield as a function of the residual gas pressure. The experimental data are shown by points, calculations by Equation (1) are shown by the solid line.

The measured X-ray yield as a function of the residual gas pressure is shown in Figure 5. The yield is given in units of the total number of counts *N* registered in the spectra at 0.066 ≤ P ≤ 6.5 mTorr.

The approximation of the experimental data was obtained by the method of least squares in the view of the linear function

*N* = 1.2 × 10^4^ × *P* + 433 (1)

where *P* is the pressure in mTorr units. The results of calculations by Equation (1) are shown by the solid line in Figure 5. One can see that the X-ray yield increases practically linearly in the pressure range from 0.066 to 6.5 mTorr. This means that the current of accelerated electrons increases practically linearly with increase of the pressure.

## 4. Discussion

Experimental research on acceleration of electrons and production of X-rays with use of the ceramic piezoelectric transformer installed in vacuum was performed. The emission of X-rays with spectral peak of characteristic radiation of the target is the clear evidence of acceleration of electrons from the high-voltage electrode of the piezoelectric transformer to the target. The returning of the charge to the high-voltage electrode of the piezoelectric transformer is demonstrated. The X-ray spectra were measured under different pressures of the residual gas in the range 0.066–18 mTorr and X-ray yield was measured in the range 0.066–6.5 mTorr. The X-ray yield increases at increasing of the residual gas pressure within two orders of magnitude and it can be approximated by the linear function (Equation (1)). The sufficient growth of the background and gas discharge were observed at increasing of the residual gas pressure above about 10 mTorr. The maximum of the accelerating voltage, the maximum energy of accelerated electrons and the maximum X-ray energy are practically independent of the residual gas pressure in our experiments.

The possibility of acceleration of electrons and production of X-rays with moderate maximum energy of 12 keV with use of ceramic piezoelectric transformer installed in vacuum has been demonstrated. This means that the transformer creates the accelerating voltage of 12 kV only. Some increasing of the accelerating voltage is possible at application of a higher-voltage piezoelectric transformer. However, radical increasing of the accelerating voltage and energy of accelerated particles can be achieved with application of the Cockroft–Walton multiplier [13] to multiply the voltage which is produced at high-voltage electrode of a piezoelectric transformer in vacuum. The multiplied accelerating voltage can be used in a small-size accelerator of higher energy.
